# Prescribed contraceptives among woman after release from prison

**DOI:** 10.1186/s40352-015-0019-1

**Published:** 2015-04-28

**Authors:** Georgina Sutherland, Megan Carroll, Nick Lennox, Stuart Kinner

**Affiliations:** 1 Melbourne School of Population and Global Health, University of Melbourne, Melbourne, VIC Australia; 2 School of Medicine, University of Queensland, Brisbane, QLD Australia; 3 Murdoch Childrens Research Institute, Melbourne, VIC Australia; 4 School of Public Health and Preventive Medicine, Monash University, Melbourne, VIC Australia

**Keywords:** Contraception, Women, Prisoners, Post-release, Record linkage

## Abstract

**Background:**

Despite the high rates of unintended and complicated pregnancy among women who have spent time in prison, little is known about their use of prescribed contraceptives post-prison release. We used a routinely-collected medicine utilisation dataset linked to a longitudinal cohort of adults released from prison to describe the dispensing of contraceptive medication.

**Findings:**

The socio-demographic profiles of women in the cohort were characteristic of justice-involved populations: they were young, poorly educated, unemployed prior to incarceration, with a very high proportion identifying as Indigenous. Dispensing claims data showed that in the six months (180 days) after release from prison contraceptive medication had been dispensed to only 19 women (7.6%).

**Conclusion:**

Our findings raise important questions about the reproductive health needs of women in prison and after their release. This requires urgent research and policy attention with a particular focus on the potential benefits of attending to women’s sexual and reproductive health care needs in prison in preparation for return to the community.

## Background

Women, in particular Indigenous women, are the fastest growing sector of the prison population in Australia (Australian Bureau of Statistics 2012). Other developed countries have noted similar rises in rates of female incarcerations (Kong and AuCoin 2008; Ministry of Justice 2012). Typically, women in the criminal justice system experience short durations of imprisonment with high rates of re-offending (Hocking et al. 2002). As a result, there has been a concomitant rise in the number of female prisoners being released back into the community. The post-release period is known to be challenging for all prisoners because of multiple and complex unmet physical, social and mental health needs (Australian Institute of Health and Welfare 2012; Cutcher et al. 2014). For women there are added complexities that relate to their pathways to prison. Previous studies have noted that exposure to childhood and adult sexual abuse, intimate partner violence and alcohol and illicit substance use put prison-involved women at greater risk of poor sexual and reproductive health outcomes including sexually transmitted infection (STI) and unintended pregnancy (Clarke et al. 2006a; Martin et al. 2012).

According to surveys of reproductive histories conducted in Australian prisons, incarcerated women have a higher number of pregnancies and become first-time mothers at a much younger age than the general birthing population (Butler et al. 2012; Indig et al. 2010; Richters et al. 2008). While there are no accurate data on either intendedness of birth or terminations in Australia, it appears that women in prison experience unintended pregnancy and terminations at substantially higher rates than the general population of child-bearing women (Butler et al. 2010; Quinn 2008). Yet, the high prevalence of sexual health problems in prisoners means most research in the area has focused on methods to reduce STI transmission, mostly conducted *within* correctional settings. Much less is known about access to forms of birth control other than condoms after release from prison: a time of heightened risk for unplanned and complicated pregnancy.

The adverse health and social consequences of unintended pregnancy for women and children are amply documented (Gipson et al. 2008), emphasising how it important it is for women to be able to plan their pregnancies. Several studies have noted that special attention is needed to ensure that the contraceptive needs of vulnerable groups are met while ensuring their choices are made freely and for themselves (Dehlendorf et al. 2010; Gold, 2014). At the national and international level, there is a lack of information about the health and reproductive needs of women post-incarceration. The ability to access contraception and plan pregnancies, as desired, is a fundamental step for women with histories of imprisonment to break the cycle of disadvantage, but a lack of population-specific data hampers efforts to intervene. In this short report, we use routinely-collected medicine utilisation data linked to a longitudinal cohort of adults released from prison to describe the prevalence of prescribed contraceptives for women in the first six months after their release.

### Study overview

Data for this paper were drawn from the Passports study, a randomised controlled trial designed to improve access to health services among adults released from prison in one state of Australia. Baseline data were collected in prison between August 2008 and July 2010 from adults incarcerated in seven correctional facilities in Queensland who were within six weeks of their expected release from prison (N = 1325). Structured baseline interviews were administered by trained research personnel in a private location in prison. Follow up telephone interviews were conducted either in the community or for those re-incarcerated, in prison, at approximately one, three and six months post release. Women were purposively over-sampled (n = 280). A more detailed description of the intervention and study can be found elsewhere (Kinner et al. 2013; Kinner et al. 2014).

### Measures

We used the baseline interviews to describe the socio-demographic characteristics and reproductive histories for women in the cohort aged between 18 and 49 years of age. We restricted our analyses to women in this age range to facilitate comparisons with population-level data on child-bearing women in Australia. The baseline survey covered a range of socio-demographic information including age, education, employment history, and relationship status prior to incarceration.

With participant consent, prison medical records were accessed to obtain information on current medication use. From this we extracted information on the use of prescribed contraception while in prison. Women were asked if they were pregnant at the time of the baseline interview.

Data from consenting female participants were linked to their Pharmaceutical Benefits Scheme (PBS) claims data post-prison release via probabilistic data linkage. Prescribed pharmaceuticals dispensed to Australian residents are subsidised through this scheme. We searched PBS claims data for the dispensing of contraceptive medications within 30, 90 and 180 days of release from prison. Prescribed contraceptives listed on the PBS included combined and progestogen-only oral contraceptive pill, progestogen-only sub-dermal implants, progestogen injection (such as Depo-Provera) and the hormonal levonorgestrel intrauterine device (IUD) known in Australia as Mirena.

Ethical clearance for the study was approved by the University of Queensland’s Behavioural and Social Sciences Ethical Review Committee and the Queensland Corrective Services Research Committee. Data linkage was approved by the Commonwealth Department of Human Services.

## Findings

Of the total sample of women in the cohort, 93% (n = 262) provided consent for their interview data to be linked to routinely-collected administrative health and medical records, including the PBS. The vast majority of these women (n = 251) were aged under 50 at the time of their baseline interview. As can be seen in Table 1, the socio-demographic characteristics of participants were consistent with the known profile of incarcerated women in Australia: they were young, poorly educated, most were unemployed prior to incarceration and one-third identified as being of Aboriginal or Torres Strait Islander descent. The average length of current incarceration was five months (M = 151 days; range 18 days to 7 years). Only two women were taking prescribed contraceptives while in prison.

**Table 1:** **Baseline socio-demographic characteristics of women <50 years (n = 251)**

**Variable**	**Percent (n)**
Age 18–24	25.5% (64)
25–49	74.5% (187)
Indigenous	35.5% (89)
< 10 years formal schooling	74.5% (187)
Unemployed	72.1% (181)
Stable relationship	46.2% (116)
Dependent children	46.9% (115)
Previous incarceration	60.5% (152)
History of sexually transmitted infection (STI)	31.1% (78)
Currently pregnant	9.5% (18)
Prescribed contraception in prison	<1% (2)

PBS claims data showed that 30 days after release from prison contraceptive medication had been dispensed to only five women (2%). At 90 days, this rate increased to approximately 4% (n = 9) and by 180 days (six months post release), 19 women (7.6%) had had contraceptives dispensed through the PBS. Around half of the women (n = 9) with PBS claims data for contraceptive medication were dispensed oral contraceptives (OC). The remainder (n = 10) were dispensed one of the long-acting reversible contraceptives (LARC).

The 19 women with records of dispensed contraceptive medication after release from prison had the same median age as all other women in the cohort (range 20–40). We did not perform tests of association between prescribed contraceptives post-prison release and socio-demographic characteristics because of the small cell frequencies, but the proportions suggest no systematic differences between the two groups. The only exception was for Indigenous status, where 17 of the 19 women who were dispensed prescribed contraceptives were non-Indigenous. Of the two women taking OC in prison, only one had filled a prescription within 180 days of release.

## Conclusions

Our study suggests that woman released from prison have alarmingly low rates of prescribed contraceptive use. In Australia, medication is the most common form of general contraception (Mazza et al. 2012). Although comparable national data are difficult to obtain, other studies using survey methods have found that around 30% to 40% of women between the ages of 18 and 49 years use OC (Richters et al. 2003; Australian Bureau of Statistics 1998). Notwithstanding that we cannot distinguish the sub-population within this cohort at risk of unintended pregnancy - those who are sexually active and not planning to become pregnant - by comparison, less than 4% of women in our cohort were dispensed OC up to six months post-prison release. Even accounting for all other types of prescribed contraceptive medication, rates of use for women in this study are around one-quarter the levels for all other Australian women in the same age bracket. The proportion of Indigenous women who were dispensed contraceptive medication post release was even smaller.

Our results, although limited in scope, add to the existing body of evidence about unmet contraceptive needs among some of the most vulnerable women living in the community. Two previous studies in Australia have shown poor uptake of contraception among women with hepatitis C (Banwell et al. 2003) and women attending drug treatment services (Black et al. 2012). Both these groups share similar profiles to incarcerated women with high rates of unplanned and high risk pregnancies. The study by Banwell et al. (Banwell et al. 2003) described contraceptive use among their cohort of women as ‘disturbingly low’ with 9% using OC. Dispensing of contraceptive medication for women in our study was even lower. These results raise important questions about the way reproductive health needs of women are addressed in prison and upon return to the community, but must be viewed in light of several study limitations.

The main set of limitations relates to our method of ascertainment and potential bias towards undercounting the number of women dispensed contraceptives post-prison release. First, we were unable to ascertain contraceptives not listed on the PBS, including copper IUDs, the vaginal ring and ‘new generation’ OC containing drospirenone. While these new formulations are described as being popular in Australia, we were unable to find reliable estimates of use in the community. Given their relative high cost, however, we are confident that in this cohort of disadvantaged women the dispensing of contraceptives that are not subsidised through the PBS does not account for the very low rates we observed. Second, dispensing of LARC is a likely underestimate given our relatively short follow up period. If women had previously been prescribed a LARC, they may not need a replacement for between three and five years, depending on the device. Nevertheless, we would not expect this undercount to substantially affect our overall estimate because the uptake of LARC methods in Australia is low, at only around 6% in the general population (Black et al. 2013). Third, we were not able to describe birth control methods that do not require a prescription, including condoms, diaphragms or the emergency contraceptive pill. Given the high rates of STI’s in this population, women in prison and after release may be encouraged and therefore more likely to use condoms as their main form of contraception. Finally, we may not have captured access to very low cost prescription contraceptives because the PBS, during our period of data collection, did not record the dispensing of medications priced below the co-payment.

Our results warrant further urgent research to explore the reasons for what appears to be very low levels of prescribed contraceptives among women after their release from prison. Women may experience multiple barriers to accessing contraception after their release from prison including not being in a position to negotiate use, the effects of alcohol or other drugs, misinformation about options, and lack of power in decision-making (Clarke et al. 2006b; Sufrin et al. 2009). For all these reasons, family planning and the provision of safe, effective and affordable contraception, according to women’s needs, should be considered a key component of health care delivered in prisons. Previous research conducted in a correctional facility in the US showed that offering women the chance to initiate birth control in prison (if they wished to do so) substantially improved uptake compared to when services were offered post-release only (Clarke et al. 2006b). This is a powerful illustration of the benefits of throughcare: care that spans from incarceration to community. With only two women using a contraceptive medication in prison in our cohort, we were unable to explore this further.

Our data suggest there is a need to improve access to fertility control methods *in prison*: a unique point of access for many high risk women. In contrast to historical coercive practices that have plagued debate about the provision of contraception to vulnerable women, we propose that prison services can provide women, who are often socioeconomically and medically marginalised when they return to the community, an opportunity to make fully informed and voluntary decisions about when or if they want children or to manage the number and spacing of children.
